# Mechanistic insights into the effects of SREBP1c on hepatic stellate cell and liver fibrosis

**DOI:** 10.1111/jcmm.15614

**Published:** 2020-07-17

**Authors:** Shengyan Su, Haimeng Tian, Xin Jia, Xiaofei Zhu, Juanjuan Wu, Yali Zhang, Yuanyuan Chen, Ziqiang Li, Yajun Zhou

**Affiliations:** ^1^ Department of Biochemistry & Molecular Biology Medical College Nantong University Nantong China; ^2^ Key Laboratory of Microenvironment and Translational Cancer Research, Medical College Nantong University Nantong China

**Keywords:** hepatic stellate cell, liver fibrosis, methionine adenosyltransferase, sterol regulatory element‐binding protein 1c

## Abstract

Sterol regulatory element‐binding protein 1c (SREBP1c) plays key roles in maintenance of hepatic stellate cell (HSC) quiescence. The present researches investigated the mechanisms underlying the effects of SREBP1c on HSCs and liver fibrogenesis by HSC‐targeted overexpression of the active SREBP1c using adenovirus in vitro and in vivo. Results demonstrated that SREBP1c exerted inhibitory effects on TAA‐induced liver fibrosis. SREBP1c down‐regulated TGFβ1 level in liver, reduced the receptors for TGFβ1 and PDGFβ, and interrupted the signalling pathways of Smad3 and Akt1/2/3 but not ERK1/2 in HSCs. SREBP1c also led to the decreases in the protein levels of the bromodomain‐containing chromatin‐modifying factor bromodomain protein 4, methionine adenosyltransferase 2B (MAT2B) and TIMP1 in HSCs. In vivo activated HSCs did not express cyclin D1 and cyclin E1 but SREBP1c down‐regulated both cyclins in vitro. SREBP1c elevated PPARγ and MMP1 protein levels in the model of liver fibrosis. The effect of SREBP1c on MAT2B expression was associated with its binding to MAT2B1 promoter. Taken together, the mechanisms underlying the effects of SREBP1c on HSC activation and liver fibrosis were involved in its influences on TGFβ1 level, the receptors for TGFβ1 and PDGFβ and their downstream signalling, and the molecules for epigenetic regulation of genes.

## 1 INTRODUCTION

Liver fibrosis is the result of chronic liver injury which causes the accumulation of excess, abnormal extracellular matrix (ECM). ECM is produced mainly by activated hepatic stellate cells (HSCs), and thus HSC activation is a key step in liver fibrogenesis.^1^

Hepatic stellate cell activation requires global reprogramming of gene expression, which must be orchestrated by key transcription regulators. Quiescent HSCs were demonstrated to be much like adipocytes and HSC transdifferentiation from quiescent to myofibroblastic cells is analogous to adipocyte‐to‐pre‐adipocyte (fibroblast) transdifferentiation.^2^ Sterol regulatory element‐binding protein 1c (SREBP1c) and peroxisome proliferator‐activated receptor γ (PPARγ), both key transcription factors for adipocyte differentiation, have also been shown to play crucial roles in inhibiting HSC activation.^2^ Ectopic transduction of SREBP1c or PPARγ causes a morphological and biochemical reversal of activated HSCs to quiescent cells in vitro.^3^, ^4^ The effect of SREBP1c seems to be more drastic and rapid as compared with that of PPARγ.^2^ These results support the hypothesis that HSCs share adipocyte phenotype and HSC quiescence is attained by the transcriptional program required for adipocyte differentiation.^2^

The mechanistic researches demonstrated the in vitro influences of PPARγ on retinyl esters, AP‐1 activity, HSC proliferation^4^ and on other transcription factor expression for adipocyte differentiation^3^ in HSCs. The in vivo studies showed that PPARγ affected NF‐kB activity in HSCs^5^ and influenced the levels of adiponectin and TGFβ in liver.^6^ The mechanisms underlying the roles of SREBP1c in liver fibrogenesis are largely undefined, and the in vivo molecular events underpinning the effects of SREBP1c on HSC activation and liver fibrogenesis have not been investigated.

Therefore, the present researches aimed to investigate the mechanisms for SREBP1c‐dependent inhibition of HSC activation and liver fibrosis in vivo and in vitro and mainly focused on the influences of SREBP1c on the protein levels of the important molecules and signalling pathways which were associated with HSC activation and liver fibrosis. As adipogentic regulation is essential for HSC quiescence while it makes hepatocyte steatotic,^7^ the studies adopted HSC‐targeted expression of the active SREBP1c by adenovirus.

## 2 MATERIALS AND METHODS

### 2.1 Materials

Thioacetamide (TAA) was purchased from Sigma (St. Louis, MO, USA). Alanine Aminotraasferase (ALT) Colorimetric Assay Kit (E‐BC‐K235), Aspartate Aminotransferase (AST) Colorimetric Assay Kit (E‐BC‐K236) and Mouse Transforming Growth Factor‐β1 (TGFβ1) ELISA Kit (E‐EL‐M0051c) were purchased from Elabscience Biotechnology Co. Ltd. (Wuhan, China).

### 2.2 HSC isolation and culture

Hepatic stellate cells were isolated from normal adult mice by in situ digestion of the liver (Animal Research Center of Nantong University, Nantong, China) as we described previously.^8^ The purity of HSC was examined by ultraviolet excited fluorescence microscope and exceeded 94%. HSCs were cultured in Dulbecco's modified Eagle's medium (DMEM) with 10% foetal bovine serum (FBS) (unless otherwise indicated) and used within 2‐10 days.

### 2.3 Western blot analysis

Western blot analyses were performed as we described previously.^8^ The used antibodies were showed in Supporting information.

### 2.4 Plasmid constructs and transient transfection assay

The methionine adenosyltransferase 2B1 (MAT2B1) promoter (−2110 to +133) was amplified from mouse genomic DNA and was inserted into SacI/XhoI sites of pGL4‐basic plasmid (Promega, Madison, WI, USA) and named as pGL4MAT2B1 (−2110)Luc. For constructing the site‐mutated MAT2B1 promoter reporter plasmid, two possible SREBP1c binding site (at −1239 bp and −1268 bp) in MAT2B1 promoter were mutated by using pGL4MAT2B1 (−2110)Luc and KOD‐Plus‐Mutagenesis Kit (TOYOBO CO, LTD., Osaka, Japan) according to the manufacturer's instructions and named, respectively, as pGL4MAT2B1 (mut1)Luc (mutation around site −1239 bp) and pGL4MAT2B1 (mut1,2)Luc (mutation around site −1239 bp and −1268 bp). The primers for construction of these plasmids were showed in Supplementary data.

Hepatic stellate cells at 70% confluence in twelve‐well plastic plates were transfected with 1 μg of pGL4MAT2B1 (−2110)Luc or pMAT2B1 (mut1) or pMAT2B1 (mut1,2) plus 0.6 μg of pSVSportSREBP1c (encoding mouse active SREBP1c, addgene, Cambrige, MA, USA) or the empty vector by using LipofectAMINE reagent (Life Technologies, New York, NY, USA). The control vector expressing Renilla luciferase (pRL‐TK; Promega) was cotransfected into the cells, and luciferase activities were quantified fluorimetrically by using the Dual‐Luciferase Reporter Assay System (Promega). The data were expressed as the ratios of Photinus to Renilla luciferase activity.

### 2.5 Electrophoretic mobility shift assay (EMSA)

EMSA assays were performed by using LightShift Chemiluminescent EMSA Kit (Pierce Biotechnology, Rockford, IL, USA) according to the manufacturer's instructions. Briefly, cultured HSCs were infected with 100 multiplicity of infection (MOI) of adenovirus AdSREBP1c (encoding mouse active SREBP1c) and incubated for 48 hours. Nuclear proteins of the HSCs were extracted by NE‐PER Nuclear and Cytoplasmic Extraction Reagents according to the manufacturer's instructions. Biotinylated DNA fragments (containing the potential SREBP1c binding site in MAT2B1 promoter) were synthesized (Life Technologies, Shanghai, China) and incubated with 5 μg protein of nuclear extract in the binding buffer at 25°C for 20 minutes. For the competition assay, the nuclear extract was pre‐incubated with 200‐fold molar excess of the unlabelled probes before addition of the labelled probe. For supershift assay, the nuclear extract was pre‐incubated with 2 μg of SREBP1 (SC‐13551, Santa Cruz, CA, USA) antibody before addition of the labelled probes. The mixtures were then subjected to electrophoresis in a 5% non‐denatureating polyacrylamide gel and then transferred onto a nylon membrane for detection by substrate working solution. The probe sequence was showed in Supporting information.

### 2.6 Chromatin immunoprecipitation (ChIP) assay

ChIP assays were carried out by using Beyotime Chip Assay Kit (P2078, Beyotime Biotechnology, Shanghai, China) according to the manufacturer's instructions. Briefly, HSCs infected with AdSREBP1c (as described in EMSA) were cross‐linked with 1% formaldehyde and incubated with SDS Lysis Buffer on ice. After the lysed cells were sonicated, the fragments of the chromatin (some for input) were incubated with the Protein A + G Agarose/Salmon Sperm DNA and underwent centrifugation. 2 μg of antibody against SREBP1 (SC‐13551, Santa Cruz) was added to the supernatant and incubated at 4°C. The Protein A + G Agarose/Salmon Sperm DNA was added to the mixture and then was centrifuged. The pellets were treated with Elution buffer, and the eluates were heated and digested with Proteinase K for purifying DNA fragments. The purified DNA fragments from the eluates and the input samples were used for PCR analysis of the fragments containing a potential SREBP1c binding site of MAT2B1 promoter. The primers for ChIP were showed in Supplementary data. The PCR products were subjected to electrophoresis in 2% agarose gel.

### 2.7 Animal studies

A liver model of TAA‐induced injury^9^ was used for animal studies. Treatment with TAA for 4 weeks (200 μg/g body weight, three times a week, by intraperitoneal injection (i.p.) led to liver fibrosis in mouse.^9^ The adenovirus AdSREBP1c (encoding mouse active SREBP1c and GFP under control of the α‐smooth muscle actin (α‐SMA) promoter) were constructed by Abm (Richmond, BC, Canada) and used for HSC‐targeted expression of the active SREBP1c in vivo. The sequence of active form of SREBP1c was from the plasmid pSVSportSREBP1c^10^ (8883, addgene). α‐SMA promoter was amplified from mouse α‐SMA gene using the primers^11^ which was shown in Suppelmentary data. This promoter of α‐SMA could be activated in HSCs, but not in hepatocytes, Kupffer cells or endothelial cells.^11^ The male C57BL/6 mice, 6 weeks old, were randomly separated into two groups (six mice/each group) and received AdSREBP1c (1 × 10^10^ pfu/mouse) or the Adcontrol (encoding GFP, Abm) by tail vein injection following 1 week of TAA treatment (200 μg/g body weight, three times a week, by i.p.) and then were treated with TAA for another 3 weeks. All the mice were killed 4 weeks after TAA treatment. Blood was harvested. The livers were fixed in 4% buffered paraformaldehyde for immunostaining analyses. The mice were given free access to water and standard chow diet and received humane care. Experiments were approved by the Institutional Animal Care and Use Committee of the University of Nantong (2012‐0031).

### 2.8 Immunostaining analysis and Sirius red staining

Double fluorescence staining was conducted for examining the respective proteins in HSCs in mouse livers. Briefly, the livers were fixed with 4% buffered paraformaldehyde. After the liver sections were blocked with normal serum, the samples were incubated with primary antibody and the respective secondary antibody (shown in Supporting information). The nuclei were stained by Hoechst 33342 (Sigma). The images were captured with the fluorescence microscope. The positive cells were counted in six randomly chosen high‐power fields at 200‐fold magnification.

For evaluating liver fibrosis, Sirius red was used to stain collagen in liver sections. Briefly, the samples were stained with picric acid‐fast green (Amresco, Solon, OH, USA) for 10 minutes and then incubated with picric acid–Sirius red (Amresco) for 1 hour. Images were captured with light microscope. The stained areas were quantified using ImageJ software (NIH, Bethesda, MD, USA).

### 2.9 Measurement of serum transforming growth factor‐β1 (TGF‐β1) and transaminases

The levels of plasma TGF‐β1, ALT and AST were evaluated by using the respective kit according to the manufacturer's instructions. Briefly, for determining the levels of plasma ALT and AST, 5 μL of plasma was incubated with 20 μL of reagent III at 37°C for 30 minutes, followed by incubation with 20 μL of reagent IV at 37°C for 20 minutes. The reactions were stopped by addition of 200 μL of reagent V. Absorbance at 510 nm was recorded and the final concentrations of the transaminases were calculated. The results expressed as U/L (international unit/L). For determining the levels of plasma TGF‐β1, 100 μL of plasma was incubated at 37°C for 90 minutes and then the solution was removed for addition of 100 μL of biotinylated antibody. The mixture was incubated for at 37°C for 60 minutes. After the process, the wells were washed for incubation with the 100 μL of HRP conjugate working solution, the 100 μL of Substrate Reagent. At last, the reaction was stopped by Stop Solution and the absorbance at 450 nm was determined. The concentrations were obtained by the standard curve, and the results were expressed as pg/mL.

#### 2.9.1 Measurement of liver hydroxyproline

The hydroxyproline levels in liver were a marker for surrogate collagen content and measured as we previous described.^12^ Hydroxyproline levels were determined by the wave length of 560 nm and results were expressed as μg/g of wet liver.

#### 2.9.2 Statistical analysis

All results were expressed as mean values ± standard deviation (SD). Statistic differences between means were evaluated using an unpaired two‐sided Student's *t* test. The comparisons of multiple treatment conditions with controls were analysed by ANOVA with the Dunnett's test for post hoc analysis. Each result was obtained from at least three independent experiments. *P* value < 0.05 was considered significant.

## 3 RESULTS

### 3.1 The effects of SREBP1c on liver fibrosis in the model of TAA‐induced liver injury

**FIGURE 1:**
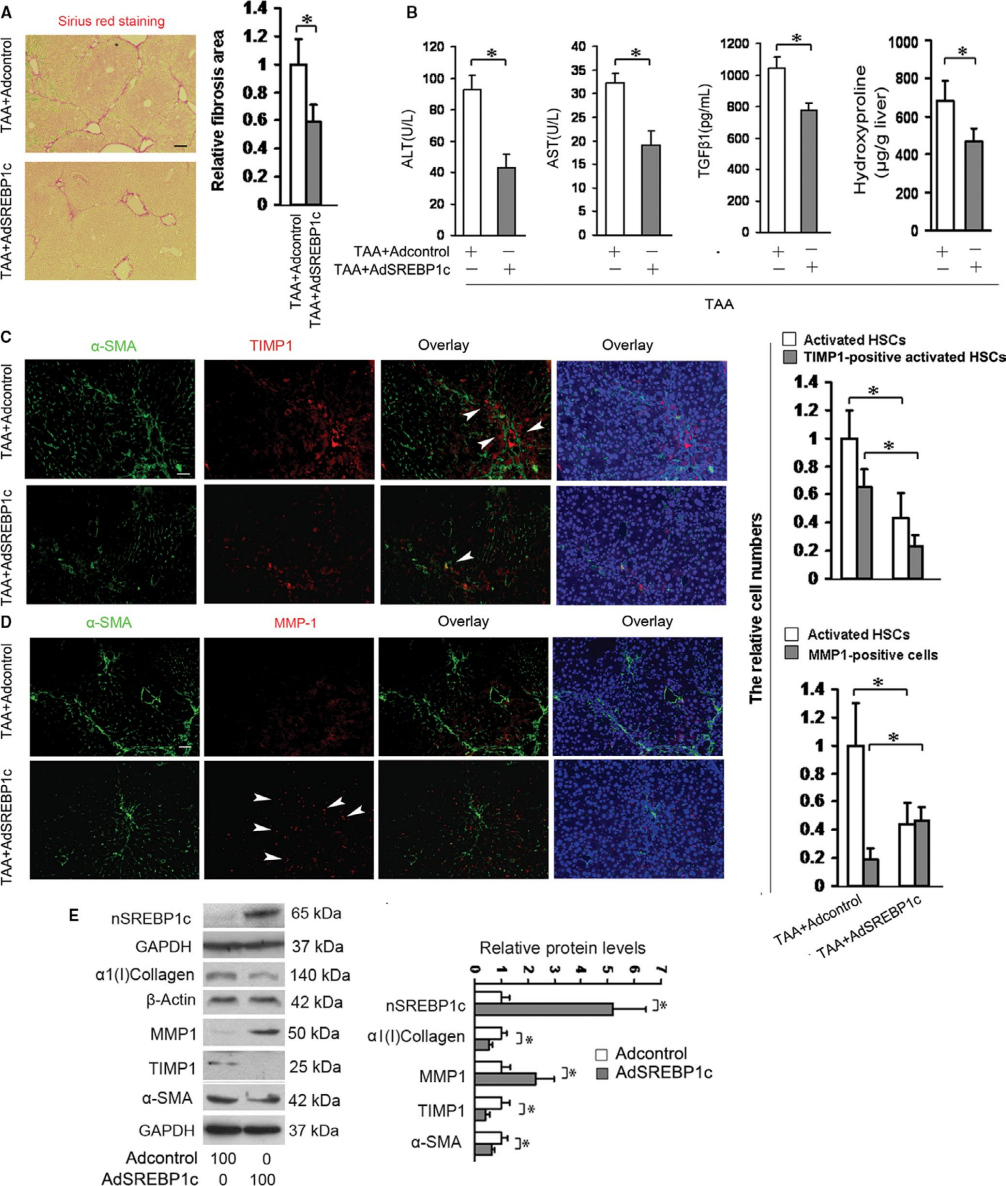
The effects of SREBP1c on liver fibrosis. A, B, Sirius red staining of the collagen and measurements of hydroxyproline, TGFβ1, ALT and AST levels. Two groups of mice (six mice/each group) were received AdSREBP1c (1 × 10^10^ pfu/mouse) or the Adcontrol by tail vein injection following one week of TAA treatment (200 μg/g body weight, three times a week, by i.p.) and then were treated with TAA for another three weeks. Sirius red staining of the liver sections (A) and measurement of hydroxyproline in the livers (B) were performed. Images were captured with light microscope. Scale bar: 100 μm. *P < 0.05. C, D, Double fluorescence staining of TIMP1 and MMP1. The liver sections from the above groups were used for double fluorescence staining of TIMP1 (C) and MMP1(D) in activated HSCs (α‐SMA). The nuclei were counterstained with Hoechst 33342. The images were captured with the fluorescence microscope. The positive HSCs were counted in six randomly chosen high‐power fields at 200‐fold magnification, and the fold changes were shown. Scale bar: 50 μm. *P < 0.05. E, Western blot analyses of the active SREBP1c (nSREBP1c), α1(I)collagen, MMP1, TIMP1 and α‐SMA (n = 3). Culture‐activated HSCs were transducted with AdSREBP1c or the Adcontrol at 100 MOI for 48 h and Western blot analyses were conducted. β‐Actin or GAPDH were used as an internal control, and the densities of the bands from Western blot were expressed as fold change relative to the respective control. *P < 0.05

To investigate the effects of SREBP1c on TAA‐induced liver fibrosis, two groups of mice were received AdSREBP1c or the Adcontrol following one week of TAA treatment and then were treated with TAA for another 3 weeks. Sirius red staining on the liver sections demonstrated that AdSREBP1c treatment significantly reduced TAA‐induced liver fibrosis (Figure 1A). The hydroxyproline levels in the liver were also evaluated and showed that AdSREBP1c treatment led to the decline in the hydroxyproline level in the liver received TAA (Figure 1B). These results indicated that HSC‐targeted overexpression of the active SREBP1c in vivo exerted an inhibitory effects on TAA‐induced liver fibrosis. We also provided the immunofluorescence for GFP to show the expression of SREBP1c (Figure S4). TGFβ1 is a major factor accelerating the progression of organ fibrosis including liver^13^ and TAA can elevate TGFβ1 level^9^ and the levels of ALT and AST^14^ in mouse. Thus, we measured the levels of plasma TGFβ1, ALT and AST in the treated mice. Results indicated that the levels of TGFβ1 decreased in the mice received AdSREBP1c as compared with that received the Adcontrol (Figure 1B). AdSREBP1c treatment also reduced the levels of ALT and AST, two indicators for liver injury (Figure 1B). Furtherly, we performed haematoxylin‐eosin staining by using the liver sections and the inflammation was induced in the liver received TAA plus Adcontrol, which was attenuated by treatment with AdSREBP1c (Figure S2).

TAA can induce TIMP1 and reduce MMP1 in mouse liver.^15^ In view of the influences of SREBP1on hydroxyproline level in the liver, we detected the expressions of MMP1 and TIMP1, the factors for regulating ECM content, in the above livers. Double fluorescence staining showed that AdSREBP1c treatment reduced TAA‐induced HSC activation as compared with the control treated with Adcontrol, which were parallel to the decrease in the number of TIMP1‐positive activated HSCs and to the increase in the number of MMP1‐positive cells in the livers received AdSREBP1c (Figure 1C,D). These data evidently indicated that the inhibitory effect of SREBP1c on HSC activation was companied with up‐regulation of MMP1 and down‐regulation of TIMP1 in the model. This result was in line with the influence of SREBP1c on collagen shown in Figure 1A. For confirming the in vivo results, culture‐activated HSCs were transducted with AdSREBP1c or the Adcontrol at multiplicity of infection (MOI) of 100^4^ for 48 hours and the protein levels of α1(I)collagen (the main component of ECM), α‐SMA (the marker for HSC activation), MMP1 and TIMP1 were detected by Western blot analyses. Results demonstrated that AdSREBP1c reduced the levels of α1(I)collagen, α‐SMA and TIMP1, and increased MMP1 level in HSCs (Figure 1E). Transduction of adenovirus at MOI of 100 was without any toxicity in activated HSCs^4^ and demonstrated an efficient transduction shown by Western blot analysis (Figure 1E). The data shown in Figure 1 indicated the inhibitory effects of SREBP1c on liver fibrosis.

### 3.2 SREBP1c reduces the protein levels of TGFβR1 and PDGFβR in HSCs

**FIGURE 2:**
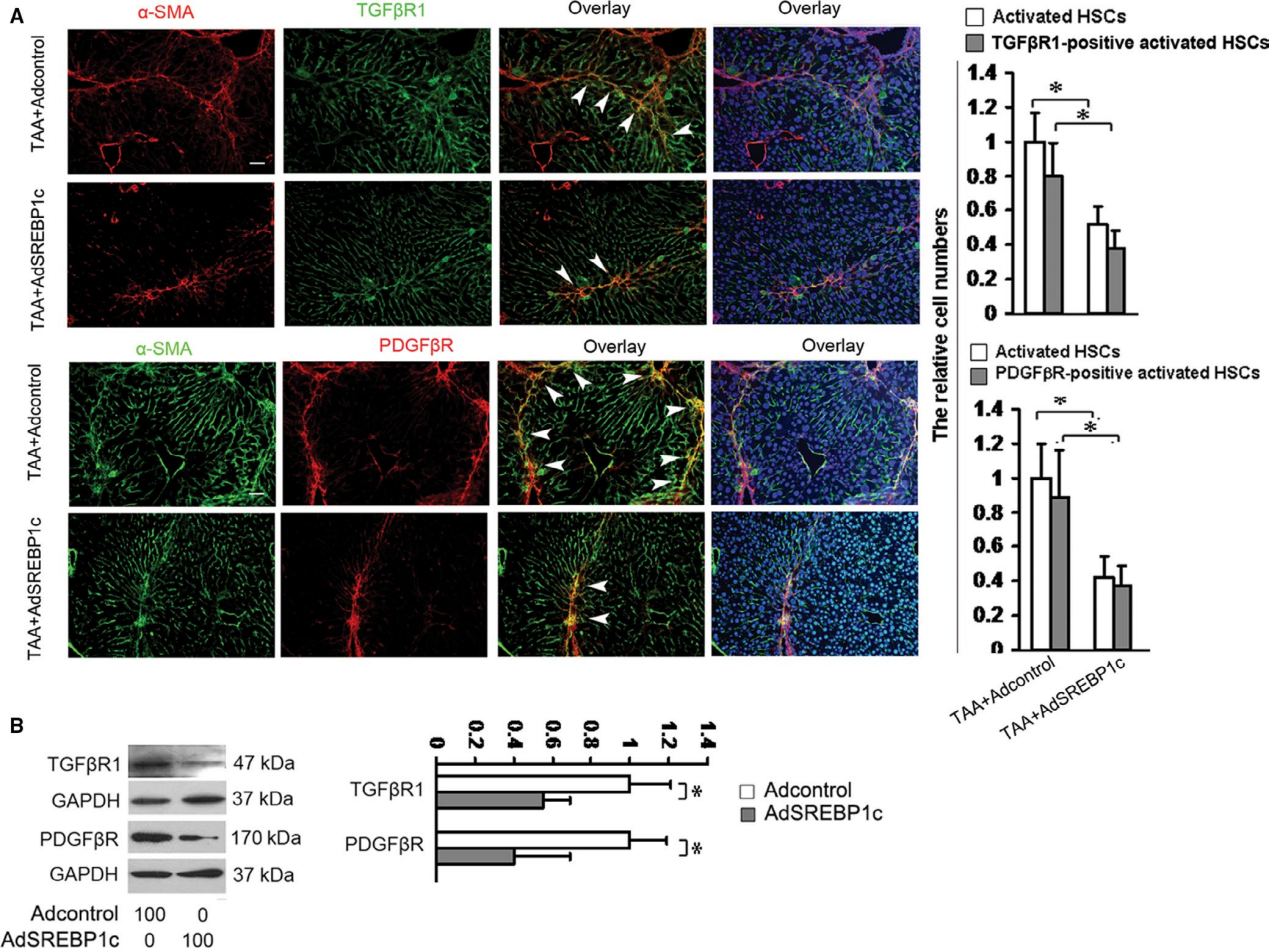
SREBP1c reduces the protein levels of TGFβR1 and PDGFβR in HSCs. A, Double fluorescence staining. Two groups of mice were treated as described in Figure 1C and double fluorescence staining was performed for evaluating the protein levels of TGFβR1 and PDGFβR in activated HSCs in the livers. The nuclei were counterstained with Hoechst 33342. The images were captured with the fluorescence microscope. The positive cells were counted in six randomly chosen high‐power fields at 200‐fold magnification, and the fold changes were shown. Scale bar: 50 μm. *P < 0.05. B, Western blot analyses of TGFβR1 and PDGFβR in activated HSCs in vitro (n = 3). Culture‐activated HSCs were treated as in Figure 1E and Western blot analyses were conducted. GAPDH were used as an internal control, and the densities of the bands from Western blot were expressed as fold change relative to the respective control. *P < 0.05

TGFβ1 and PDGFβ are identified as the most important cytokines for inducing HSC activation and liver fibrosis.^16^ In the process of liver fibrogensis, both cytokines levels increase in the liver, interact with HSCs and stimulate the expressions of their receptors (TGFβR1 and PDGFβR) in HSCs, leading to HSC activation and ECM expression.^16^ Thereby, the effects of SREBP1c on TGFβR1 and PDGFβR in HSCs were detected in the livers of TAA‐induced liver injury. Two groups of mice were treated as described in Figure 1C, and the third group of mice was received vehicle plus Adcontrol (without TAA, negative control). The livers were used for double fluorescence staining of TGFβR1 and PDGFβR in HSCs. Results showed that TAA‐induced HSC activation was followed by the increase in the number of TGFβR1‐positive and PDGFβR‐positive HSCs (Figure 2A). The activated HSCs and TGFβR1‐positive or PDGFβR‐positive HSCs could not detected in the livers received vehicle plus Adcontrol (Figure S1). These results suggested that SREBP1c affected the expressions of the most important factor receptors in HSCs. We also examined the effects of SREBP1c on both receptors in vitro. Cultured HSCs were treated as in Figure 1E and Western blot analysis showed SREBP1c‐induced decreases in the TGFβR1 and PDGFβR in HSCs in vitro (Figure 2B). These data in Figure 2 indicated that SREBP1c could reduce the protein levels of TGFβR1 and PDGFβR in HSCs in vivo and in vitro.

### 3.3 SREBP1c intererupts the signalling pathways of Smad3 and Akt1/2/3, but has no effect on ERK1/2 pathway in HSCs

**FIGURE 3:**
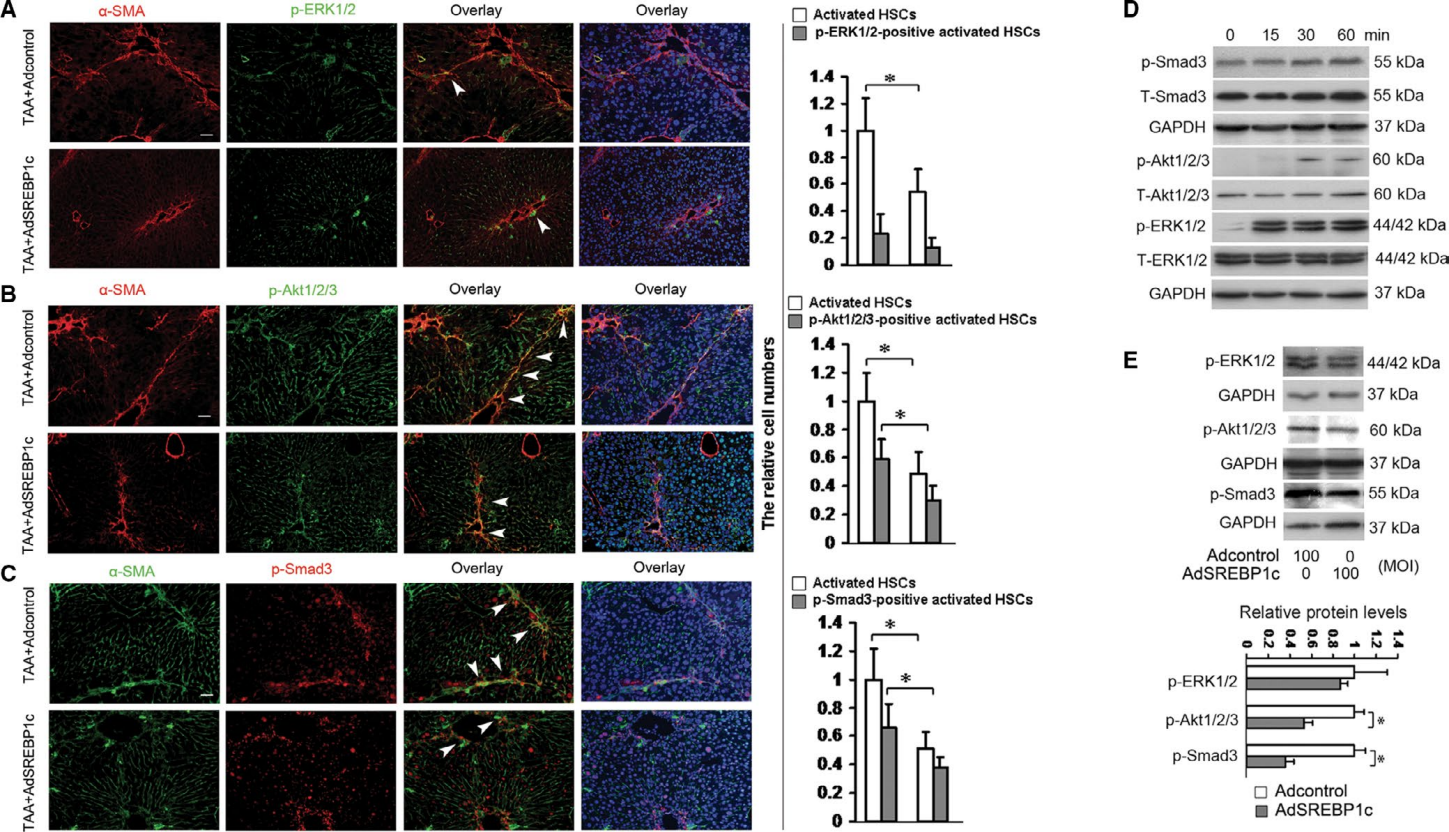
SREBP1c interrups the phosphorylations of Akt1/2/3, and Smad3, but not ERK1/2 in HSCs. A‐C, Double fluorescence staining. Two groups of mice were treated as described in Figure 1C and double fluorescence staining of the liver was performed for detecting the phosphorylations of ERK1/2 (A), Akt1/2/3 (B) and Smad3 (C) in activated HSCs. The nuclei were counterstained with Hoechst 33342. The images were captured with the fluorescence microscope. The positive HSCs were counted in six randomly chosen high‐power fields at 200‐fold magnification, and the fold changes were shown. Scale bar: 50 μm. *P < 0.05. D, E, Western blot analyses of the phosphorylations of ERK1/2, Akt1/2/3, Smad3 in activated HSCs in vitro (n = 3). Serum‐starved HSCs were incubated with the medium containing 5% of FBS for the indicated different time periods, and the phosphorylations of Smad3, Akt1/2/3 and ERK1/2 were examined by Western blot analyses (D). Another group of cultured HSCs HSCs were transducted with 100 MOI of AdSREBP1c or the Adcontrol for 24 h and then underwent serum‐starvation for 24 h before incubation with 5% of FBS for 30 min. Western blot analyses were performed (E). GAPDH were used as an internal control and the densities of the bands from Western blot were expressed as fold change relative to the respective control. *P < 0.05

TGFβR1 and PDGFβR induce several of signalling pathways such as the classic pathways of Smad, Akt and ERK1/2.^16^ Thereby, we examined in vivo effects of SREBP1c on these pathways which are associated with promotion of HSC activation, HSC proliferation and ECM syntheses.^16^ We used the same livers as in described in Figure 1C and the livers treated with vehicle plus Adcontrol (without TAA, negative control) for double fluorescence staining. In livers with vehicle plus Adcontrol (without TAA), the phosphorylated Smad3‐positive, phosphorylated Akt1/2/3‐positive and phosphorylated ERK1/2‐positive cells could not be detected (Figure S1) and treatment with TAA plus Adcontrol could not evidently lead to ERK1/2 phosphorylation in activated HSCs, which was not affected by AdSREBP1c treatment (Figure 3A). By contrast, TAA‐induced HSCs activation were accompanied with the increase in the number of phosphorylated Smad3‐positive, phosphorylated Akt1/2/3‐positive, which were inhibited by AdSREBP1c treatment (Figure 3B,C). These data suggested that SREBP1c had negative effects on at least the signalling pathways of Smad3 and Akt1/2/3 in HSCs in vivo. We further examined the results in vitro. Firstly, serum‐starved HSCs were incubated with the medium containing 5% of FBS for different time periods and Western blot analyses indicated that 30 minutes of FBS stimulation led to the phosphorylations of Smad3, Akt1/2/3 and ERK1/2 (Figure 3D). Next, HSCs were transducted with AdSREBP1c or the Adcontrol and then underwent serum‐starvation for 24 hours before incubation with 5% of FBS for 30 minutes. Figure 3E revealed the inhibitory effects on SREBP1c on the phosphorylations of Smad3 and Akt1/2/3 but not the phosphorylation of ERK1/2, suggesting that SREBP1c inhibited the signalling pathways of Smad3 and Akt1/2/3 but not ERK1/2 pathway in HSCs in vitro. Based on the in vivo and in vivo results, SREBP1c could interrupt the signalling pathways of Smad3 and Akt1/2/3 in HSCs in vivo.

### 3.4 SREBP1c cannot influence the protein levels of cyclin D1 and cyclin E1 in activated HSCs in vivo but not in vitro

**FIGURE 4:**
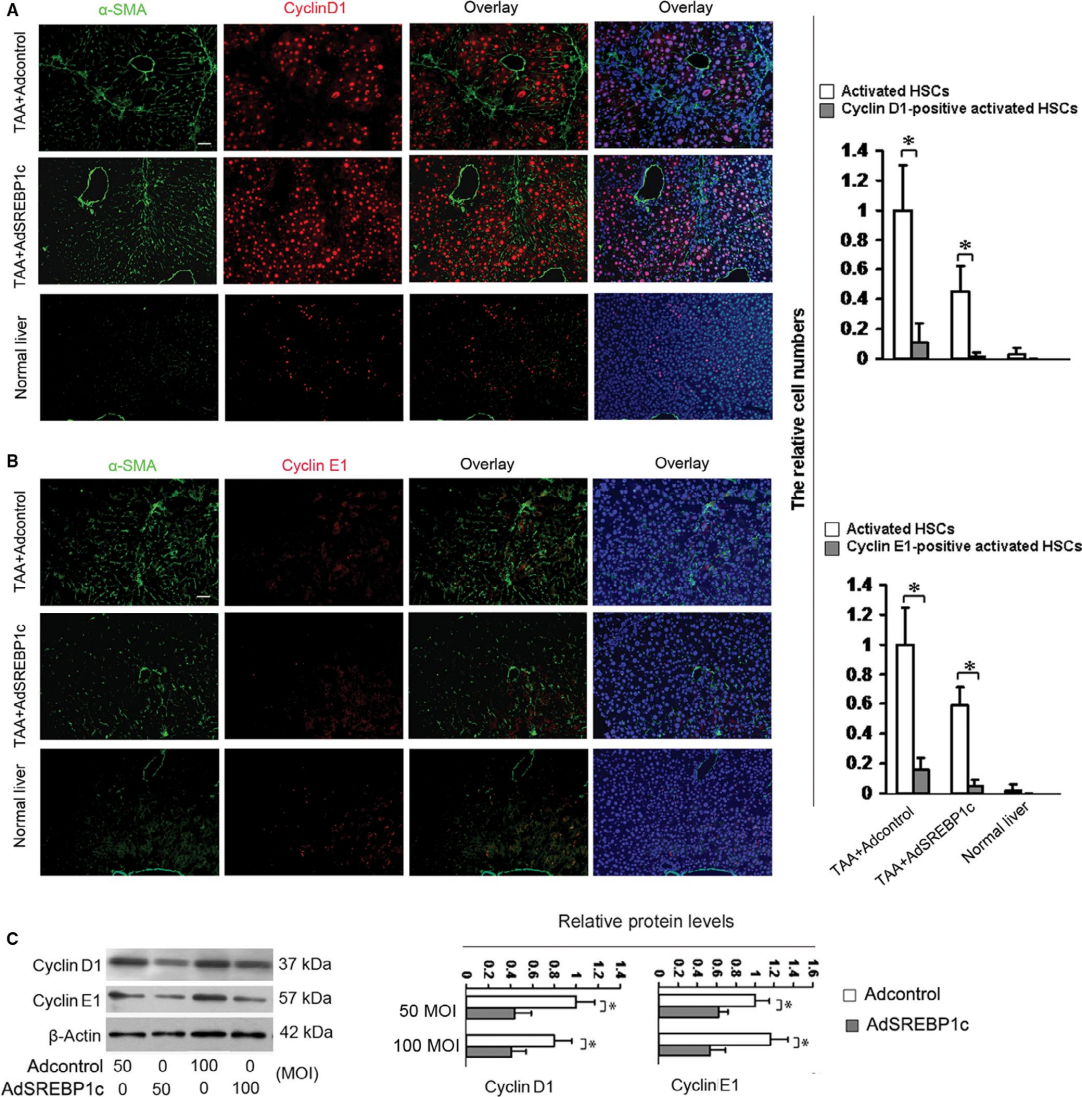
SREBP1c cannot influence the protein levels of cyclin D1 and cyclin E1 in activated HSCs in vivo but not in vitro. A, B, Double fluorescence staining. Two groups of mice were treated as described in Figure 1C and double fluorescence staining of the above liver sections or the normal liver sections was conducted for examining cyclin D1 (A) and cyclin E1 (B) in activated HSCs. The nuclei were counterstained with Hoechst 33342. The images were captured with the fluorescence microscope. The positive HSCs were counted in six randomly chosen high‐power fields at 200‐fold magnification, and the fold changes were shown. Scale bar: 50 μm. *P < 0.05. C, Western blot analyses of cyclin D1 and cyclin E1 in activated HSCs in vitro (n = 3). Culture‐activated HSCs were transducted with AdSREBP1c or the Adcontrol at 50 or 100 MOI for 48 h and cyclin D1 and cyclin E1 were detected by Western blot analyses. β‐Actin was used as an internal control, and the densities of the bands from Western blot were expressed as fold change relative to the respective control. *P < 0.05

The activation of Akt1/2/3 pathway contributes to HSC proliferation.^16^ Thus, we used the same livers as in Figure 1C and the livers treated with vehicle plus Adcontrol (without TAA, negative control) for examining in vivo effects of SREBP1c on the protein levels of cyclin D1 and cyclin E1, the key molecules for cell proliferation, in activated HSCs. Unexpectedly, double fluorescence staining demonstrated that cyclin D1 and cyclin E1 were barely detected in the activated HSCs (Figure 4A,B). The fluorescence for cyclin D1 was strong in other type of cells in the livers of TAA‐induced injury (Figure 4A), and the fluorescence for cyclin E1 was faint in the same livers (Figure 4B). AdSREBP1c treatment had no effects on both cyclin D1 and cyclin E1 in the activated HSCs in the livers. These results promoted us to examine the expressions of cyclin D1 and cyclin E1 in mouse normal livers. Double fluorescence staining showed that the cyclin D1‐positive or cyclin E1‐positive cells were very few (Figure 4A,B). To test the results in vitro, culture‐activated HSCs were transducted with AdSREBP1c or the Adcontrol at MOI of 50 or 100 for 48 hours^4^ and cyclin D1 and cyclin E1 were detected by Western blot analyses. Figure 4C showed that different doses of AdSREBP1c reduced the protein levels of both cyclins as compared with the respective control, suggesting the inhibitory effects of SREBP1c on the levels of cyclin D1 and cyclin E1 in activated HSCs in vitro.

### 3.5 SREBP1c reduces the protein levels of Bromodomain protein 4 (BRD4) and MAT2B and increases the protein level of PPARγ in HSCs

**FIGURE 5:**
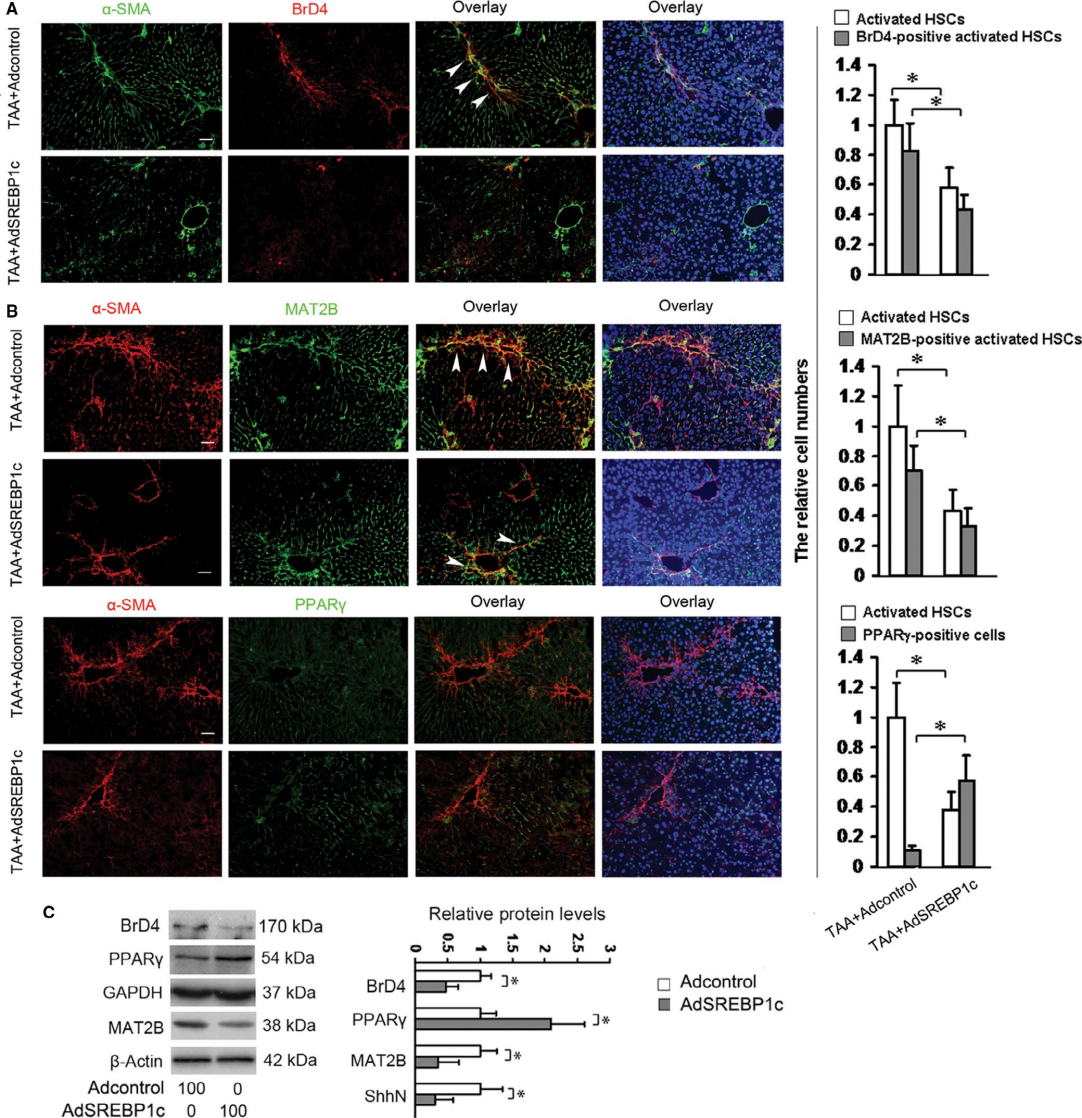
SREBP1c reduces the protein levels of BRD4 and MAT2B and increases the protein level of PPARγ in HSCs. A, B, Double fluorescence staining. Two groups of mice were treated as described in Figure 1C, and double fluorescence staining was performed for detecting the proteins of BrD4, MAT2B and PPARγ in HSCs in the livers. The nuclei were counterstained with Hoechst 33342. The images were captured with the fluorescence microscope. The positive cells were counted in six randomly chosen high‐power fields at 200‐fold magnification and the fold changes were shown. Scale bar: 50 μm. *P < 0.05. C, Western blot analyses of BRD4, MAT2B and PPARγ in activated HSCs in vitro (n = 3). Culture‐activated HSCs were treated as in Figure 1E and Western blot analyses were conducted. β‐Actin or GAPDH were used as an internal control and the densities of the bands from Western blot were expressed as fold change relative to the respective control. *P < 0.05

BrD4, the epigenetic reader,^17^ regulates pro‐fibrotic gene expression in various tissues^18^ including liver.^19^ MAT2B is associated with DNA methylation, an epigenetic mechanism for regulation of gene, and essential for HSC activation.^20^ Epigenetic regulation of gene expression is one of the important mechanisms for HSC activation.^21^ SREBP1c is a key transcription factors for inhibiting HSC activation.^2^ Hence, we tested the effects of SREBP1c on BRD4, MAT2B and PPARγ (another key transcription factor for inhibiting HSC activation) by using the livers as described in Figure 1C and the livers treated with vehicle plus Adcontrol (without TAA). Double fluorescence staining revealed that the BrD4‐positive and MAT2B‐positive cells could not be detected and PPARγ positive‐cells appeared in the livers treated with vehicle plus Adcontrol (Figure S1). TAA treatment increased the number of activated HSCs and the number of BrD4‐positive and MAT2B‐positive activated HSCs, which were reduced by AdSREBP1c treatment (Figure 5A). In contrast, PPARγ‐positive cells could not be detected in the livers treated with TAA plus Adcontrol whereas PPARγ positive‐cells appeared in the liver treated with TAA plus AdSREBP1c (Figure 5B). For the in vitro experiments, cultured HSCs were infected by AdSREBP1c or the Adcontrol as described in Figure 1E and Western blot analysis indicated that AdSREBP1c reduced BRD4 and MAT2B levels and increases PPARγ level (Figure 5C). Considering the in vivo and in vitro results, SREBP1c could reduce the protein levels of BRD4 and MAT2B and increase the protein level of PPARγ in HSCs in vivo.

### 3.6 SREBP1c suppresses MAT2B1 promoter activity by binding to MAT2B1 promoter at the site around—1239 bp

**FIGURE 6:**
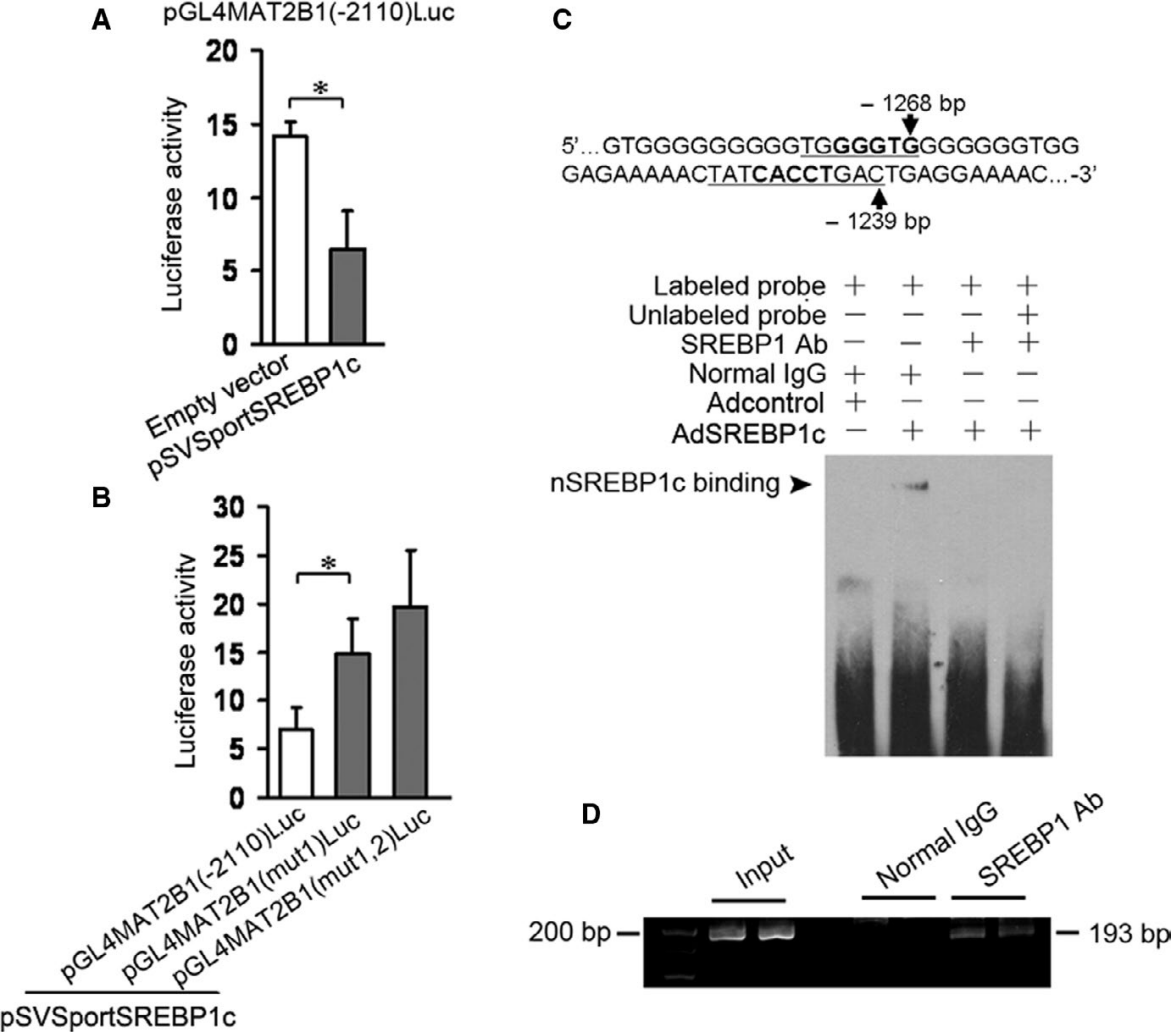
SREBP1c suppresses MAT2B1 promoter activity by binding to MAT2B1 promoter at the site around—1239 bp. A, B, Transfection assay for analysis of MAT2B1 promoter activity. Cultured HSCs were transfected with 1 μg of pGL4MAT2B1 (−2110)Luc or pMAT2B1 (mut1) or pMAT2B1 (mut1,2) plus 0.6 μg of pSVSportnSREBP1c or the empty vector. The control vectors expressing Renilla luciferase were cotransfected into the cells. The HSCs were incubated for 24 h. Luciferase activity assay was conducted. The data were expressed as the ratios of Photinus to Renilla luciferase activity. *P < 0.05. C, D, EMSA and ChIP assay for confirming SREBP1c binding to MAT2B1 promoter. Cultured HSCs were infected by 100 MOI of AdSREBP1c or the Adcontrol and incubated for 48 h. The nuclear extracts were used for EMSA (C) or the purified DNA fragments from HSCs infected with AdSREBP1c were used for PCR of the fragments (193 bp) containing the possible SREBP1c binding site in MAT2B1 promoter (D). The PCR products were subjected to electrophoresis in 2% agarose gel (D). The representative results were shown from three independent experiments

As demonstrated above, SREBP1c affected the protein levels of MAT2B. Thereby, software BioBase was used for predicting whether their promoters harbored SREBP1c binding sites and MAT2B1 promoter was found to possess two possible binding sites (around –1239 bp and –1268 bp) for SREBP1c. Based on the prediction, HSCs were cotransfected with pGL4MAT2B1(–2110)Luc plus pSVSportSREBP1c or the empty vector. Luciferase assay showed that pSVSportSREBP1c reduced MAT2B1 promoter activity (Figure 6A). Furtherly, pGL4MAT2B1(mut1)Luc (mutantion around –1239 bp) or pGL4MAT2B1(mut1,2)Luc (mutantion around –1239 bp and –1268 bp) or the pGL4MAT2B1(–2110)Luc were cotransfected into HSCs with pSVSportSREBP1c. Luciferase assay indicated that the effect of pSVSportSREBP1c on the mut1‐MAT2B1 promoter activity was attenuated (Figure 6B) and, as compared with the luciferase activity in HSCs transfected with pGL4MAT2B1(mut1)Luc, pSVSportSREBP1c had no effect on the luciferase activity in HSCs transfected with pGL4MAT2B1(mut1,2)Luc, suggesting that the site around –1239 bp but not –1268 bp might be the SREBP1c binding site. The possible SREBP1c binding site was confirmed by EMSA. Figure 6C demonstrated that the probe containing the site around –1239 bp bound to the nuclear SREBP1c encoded by AdSREBP1c. For further confirming the result, HSCs infected with AdSREBP1c were used for ChIP assay. Results showed that the bands of PCR products from the DNA fragments (containing SREBP1c binding site) immunoprecipitatied by SREBP1 antibody appeared (Figure 6D). These data in Figure 6 pointed to that SREBP1c suppressed MAT2B1 promoter activity by binding to MAT2B1 promoter at the site around—1239 bp. In view that MAT2B affects the DNA methylation,^20^ we also examined the effect of SREBP1c on DNA methylation in cultured HSCs. Results indicated that HSCs infected with AdSREBP1c led to the increase in global DNA methylation in HSCs as compared with the control (received Adcontrol) (Figure S3). Thus, MAT2B might mediate the influence of SREBP1c on DNA methylation in HSCs.

## 4 DISCUSSION

SREBP1c is a key transcription factor for maintaining HSCs in a quiescent state.^2^, ^3^ The molecular events underlying the roles of SREBP1c in maintaining quiescent HSCs are largely unknown. The present in vivo and in vitro researches demonstrated that SREBP1c exerted inhibitory effect on TAA‐induced liver fibrosis and down‐regulated TGFβ1 level. It reduced the protein levels of TIMP1, TGFβR1, PDGFβR, BrD4 and MAT2B in HSCs, and increased the protein levels of MMP1 and PPARγ in liver. Moreover, SREBP1c interrupted the classic signalling pathways of Smad3 and Akt1/2/3 but not ERK1/2 in HSCs. Unexpectedly, activated HSCs did not express both cyclin D1 and cyclin E1 in vivo whereas SREBP1c down‐regulated cyclin D1 and cyclin E1 in HSCs in vitro. The effect of SREBP1c on MAT2B expression was associated with its binding to MAT2B1 promoter at the site around –1239 bp and thus suppressed the promoter activity.

Hepatic stellate cell activation, a process of HSC transdifferentiation from quiescent to myofibroblastic cells, is a crucial step in liver fibrogenesis.^1^ The global reprogramming of HSC gene expression is required for HSC activation, which is orchestrated by key transcription regulators. SREBP1c and PPARγ, two key transcription factors for adipocyte differentiation, were demonstrated to play a crucial role in inhibiting HSC activation.^2^, ^3^, ^4^ The present studies showed the influences of SREBP1c on both receptors for TGFβ1 and PDGFβ, the most important cytokines for inducing HSC activation and liver fibrosis,^16^ and on their downstream signalling pathways of Smad3 and Akt1/2/3 in HSCs. Smad3 is the crucial signalling molecule for the pathway of TGFβ1, the potent factor in stimulating ECM syntheses.^13^ PDGF is the most potent mitogen towards HSCs and activates at least the PI3K/pAkt1/2/3 pathway, leading to HSC proliferation.^16^ Thereby, SREBP1c‐induced inhibition of HSC activation and the declines in ECM levels directly correlated with its influences on both receptors for TGFβ1 and PDGFβ and on the downstream Smad3 and pAkt1/2/3 pathways.

ERK1/2 signalling is a classic pathway for promoting cell proliferation and activated HSCs demonstrated strong proliferation ability, but the increases in phosphoylated ERK1/2 levels were not evident in TAA‐induced activated HSCs in vivo. In HSCs, SREBP1c affected neither FBS‐induced phosphoylated ERK1/2 in vitro nor ERK1/2 pathway in liver of TAA‐induced injury. Concomitantly, cyclin D1, a master regulator for promoting cell proliferation,^22^ could not be detected in activated HSCs but the strong staining of cyclin D1 in other types of cells was demonstrated in the same livers. Because of the behaviour of cyclin D1, cyclin E1 was examined and showed a weak staining in activated HSCs in the same livers. Interestingly, cyclin D1 and cyclin E1 play crucial roles in promoting cell proliferation^22^ and one remarkable feature of the activated HSC is the strong ability of proliferation, but the activated HSCs did not express cyclin D1 and cyclin E1 in the model of liver fibrosis. AdSREBP1c treatment had no effects on cyclin D1 or cyclin E1 in HSCs in the in vivo model but reduce both protein levels in HSCs in vitro. It was showed that cyclin E1 expression in HSCs was maximal when HSCs started to proliferate, but decreased after the cells transdifferentiated into myofibroblasts.^23^ Cyclin D1 and cyclin A were showed to decline in HSCs following 4 weeks of dimethylnitrosamine‐induced liver injury.^24^ These data were consistent with the low protein levels of cyclin E1 and cyclin D1 in activated HSCs in TAA‐induced liver fibrosis. In activated HSCs in vivo, the decreases in the protein levels of cyclin E1 and cyclin D1 seemed to be in line with the weak activation of ERK1/2, an important pathway for promoting cell proliferation. These results suggested a possibility that in vivo the activated HSCs might reduce the proliferation ability and waken the potential for synthesizing ECM. This possibility was supported by the evidence that TGFβR level was increased in TAA‐induced activated HSCs in vivo as TGFβ is the most potent factor for ECM synthesis in activated HSCs.^13^ The difference between the expressions of cyclin E1 and cyclin D1 in the livers of TAA‐induced injury was that cyclin D1 but not cyclin E1 was showed a high protein levels in other types of cells in the liver.

Notably, the activated HSCs expressed high levels of PDGFβR in the liver model. Considering the most potent mitogen PDGF for HSCs^25^ and the low protein levels of activated ERK1/2, cyclin D1 and cycln E1 in activated HSCs in vivo, a new question emerged: what were the roles of PDGF in vivo in activated HSCs of high level PDGFβR? A possible explanation might be the change of the role of PDGF in activated HSCs in vivo, which need to be validated.

Given our results that SREBP1c induced the declines in the protein levels of BrD4 and MAT2B in activated HSCs, SREBP1c might be involved in epigenetic regulation of the expressions of the genes associated with liver fibrogenesis. BrD4 is identified as the epigenetic reader^17^ and has been clearly shown to regulate pro‐fibrotic gene expression in liver.^19^ It associates with acetyl‐histone H3K27‐marked enhancers for a variety of pro‐fibrotic genes in HSCs and is suggested to be a novel therapeutic target for liver fibrosis.^19^ MAT2B is a regulatory subunit of MATII which catalyses the biosynthesis of S‐adenosylmethionine (SAMe), the principle biological methyl donor.^26^ It was shown that the remodelling of the DNA methylation was involved in HSC activation,^21^ and MAT2B was essential for HSC activation.^20^ Therefore, it seemed reasonable that SREBP1c also inhibit HSC activation by influencing BrD4 or MAT2B‐mediated epigenetic regulation of the gene expressions in HSCs. The effect of SREBP1c on DNA methylation in HSCs supported the notion.

As MAT2B promoter was predicted to contain two possible binding sites for SREBP1c, the further experiments revealed that one of two possible binding sites was SREBP1c binding site and SREBP1c‐induced decrease in MAT2B expression was correlated with its inhibiting MAT2B promoter by binding to the site.

PPARγ could elevate the levels of active SREBP1c in cultured HSCs^3^ and our results indicated that the active SREBP1c increased PPARγ protein level in HSCs in vivo and in vitro, suggesting that both key transcription factors for controlling HSC activation affected each other interactively and the synergies of them inhibited HSC activation.

In conclusions, the researches revealed the molecular events underlying the inhibitory effects of SREBP1c on HSC activation and liver fibrosis in vivo and in vitro. The mechanisms were associated with the effects of SREBP1c on the TGFβ1 levels, the most important receptors and their downstream signalling pathways, ECM syntheses and the molecules for epigenetic regulation of genes, providing new insights into the liver fibrogenesis.

## CONFLICT OF INTEREST

The authors declare no conflict of interest.

## AUTHOR CONTRIBUTION

**Shengyan Su:** Conceptualization (supporting); Formal analysis (equal); Investigation (lead); Resources (lead); Visualization (lead); Writing‐review & editing (supporting). **Haimeng Tian:** Data curation (supporting); Investigation (equal); Resources (supporting); Validation (equal); Writing‐review & editing (supporting). **Xin Jia:** Conceptualization (supporting); Formal analysis (supporting); Investigation (equal); Writing‐review & editing (supporting). **Xiaofei Zhu:** Investigation (equal); Writing‐review & editing (supporting). **Juanjuan Wu:** Investigation (supporting); Writing‐review & editing (supporting). **Yali Zhang:** Investigation (supporting); Writing‐review & editing (supporting). **Yuanyuan Chen:** Investigation (supporting). **Ziqiang Li:** Investigation (supporting). **Yajun Zhou:** Conceptualization (equal); Data curation (equal); Formal analysis (equal); Funding acquisition (lead); Investigation (supporting); Methodology (lead); Project administration (lead); Resources (equal); Supervision (lead); Writing‐original draft (lead); Writing‐review & editing (equal).

## Supporting information

Supplementary Material

## Data Availability

The data that support the findings of this study are available from the corresponding author upon reasonable request.
