# Supplementary material for: Mechanistic insights into the effects of SREBP1c on hepatic stellate cell and liver fibrosis
Source: J Cell Mol Med. 2020 Jul 17;24(17):10063–74. doi: 10.1111/jcmm.15614 (PMC7520338; doi:10.1111/jcmm.15614)
Supplement: Supplementary file 1 — Supplementary Material [file JCMM-24-10063-s001.doc]

**Appendix S1**

**1. The used antibodies for Western blot analysis**

The primary antibodies were as follows: Cyclin D1 (1:1000, ab134175, Abcam, MA, USA); Cyclin E1 (1:1000, sc-377100, Santa Cruz, CA, USA); SREBP1c (1:2000, SC-367, Santa Cruz, CA, USA); α1(I)collagen (1:2000, SC-293182, Santa Cruz, CA, USA); α-SMA (ab124964, 1:2000, Abcam, MA, USA); Bromodomain protein 4 (BRD4) (1:1000, ab128874, Abcam, MA, USA); MMP1 (1:500, BS1229, Bioworld Technology, Inc. MN, USA); TIMP1 (1:500, ab61224, Abcam, MA, USA); TGFβR1 (1:500, sc-101574, Santa Cruz, CA, USA); PDGFβR (1:1000, ab32570, Abcam, MA, USA); PPARγ (1:1000, sc-271392, Santa Cruz, CA, USA); methionine adenosyltransferase 2B (MAT2B) (1:1000, sc-390586, Santa Cruz, CA, USA ); phospho-Smad3 (1:1000, 9520, Cell signaling technology, Inc. MA, USA); Smad3 (1:1000, ab40854, Abcam, MA, USA); phospho-Akt1/2/3 (1:500, sc-514032, Santa Cruz, CA, USA); Akt1/2/3 (1:500, SC-377556, Santa Cruz, CA, USA); phospho-ERK1/2 (1:2000, sc-81492, Santa Cruz, CA, USA); ERK1/2 (1:2000, SC-135900, Santa Cruz, CA, USA); β-Actin (1:2000, sc-47778, Santa Cruz, CA, USA); GAPDH (1:2000, sc-365062, Santa Cruz, CA, USA). Horseradish peroxidase-conjugated secondary antibody (1:4000, Cell signaling technology, Inc. MA, USA). ECL detection system (Pierce Biotechnology, Rockford, lL, USA).

**2. The primers for construction of pGL4MAT2B1 (-2110)Luc.**

(F) SacI 5′-CGAGCTCGGCAATTCAAGACTCGGTGGC-3′;

(R) XhoI 5′-CCGCTCGAGCGGTCTTTGCGCCGCTTCAGT-3′.

**3. The primers for construction of pGL4MAT2B1 (mut1) and pGL4MAT2B1 (mut1,2).**

Around site -1239 bp:

(F) TCCCTGACTGAGGAAAACTTTTCCC

(R) ATAGTTTTTCTCCCACCCCCCCAC

(tatCACCTgac were mutated into tatTCCCTgac)

Around site -1268 bp:

(F) TTGTGGGGGGGTGGGAGAAAAAC

(R) CACCCCCCCCCACTAAAGAATTT

(tgGGGTG were mutated into tgTTGTG)

**4. The probe sequence for EMSA.**

5’-gagaaaaacTATCACCTGACtgaggaaaac-3’

**5. The primers for ChIP assay.**

(F) 5’-GCCTTTGTTTGCTCTTCCA-3’

(R) 5’-CGTGACCACTGCCTGAATT-3’

**6**.**The primers for amplifying mouse α-SMA promoter.**

(F) 5’-CGGGGTACCCCGCACCATAAAACAAGTGCATGAG-3’

(R) 5’-CCGCTCGAGCGGGCTGGAGCAGCGTCTCAGG-3’.

**7. The used antibodies for** **Immunostaining analysis.**

The primary antibody were as follows: TGFβR1 (1:15, sc-101574, Santa Cruz, CA, USA); PDGFβR (1:30, ab32570, Abcam, MA, USA); BrD4 (1:200, ab128874, Abcam, MA, USA); MAT2B (1:20, sc-390586, Santa Cruz, CA, USA ); PPARγ (1:15, sc-271392, Santa Cruz, CA, USA); phospho-ERK1/2 (1:50, SC-81492, Santa Cruz, CA, USA); phospho-Akt1/2/3 (1:20, sc-514032, Santa Cruz, CA, USA); phospho-Smad3 (1:20, 9520, Cell signaling technology, Inc. MA, USA); Cyclin D1 (1:20, ab134175, Abcam, MA, USA); Cyclin E1 (1:20, BS1058, Bioworld Technology, Inc. MN, USA); MMP1 (1:20, BS1229, Bioworld Technology, Inc. MN, USA); TIMP1 (1:20, ab61224, Abcam, MA, USA); or α-SMA (1:250, ab124964, Abcam, MA, USA; 1:20, sc-32251, Santa Cruz, CA, USA). Alexa Fluor® 594- or Alexa Fluor® 488-conjugated secondary antibody (1:200, ab150084, ab150117, Abcam, MA, USA).

**8. Fig. S1. Double fluorescence staining of the livers** **received vehicle plus Adcontrol**.


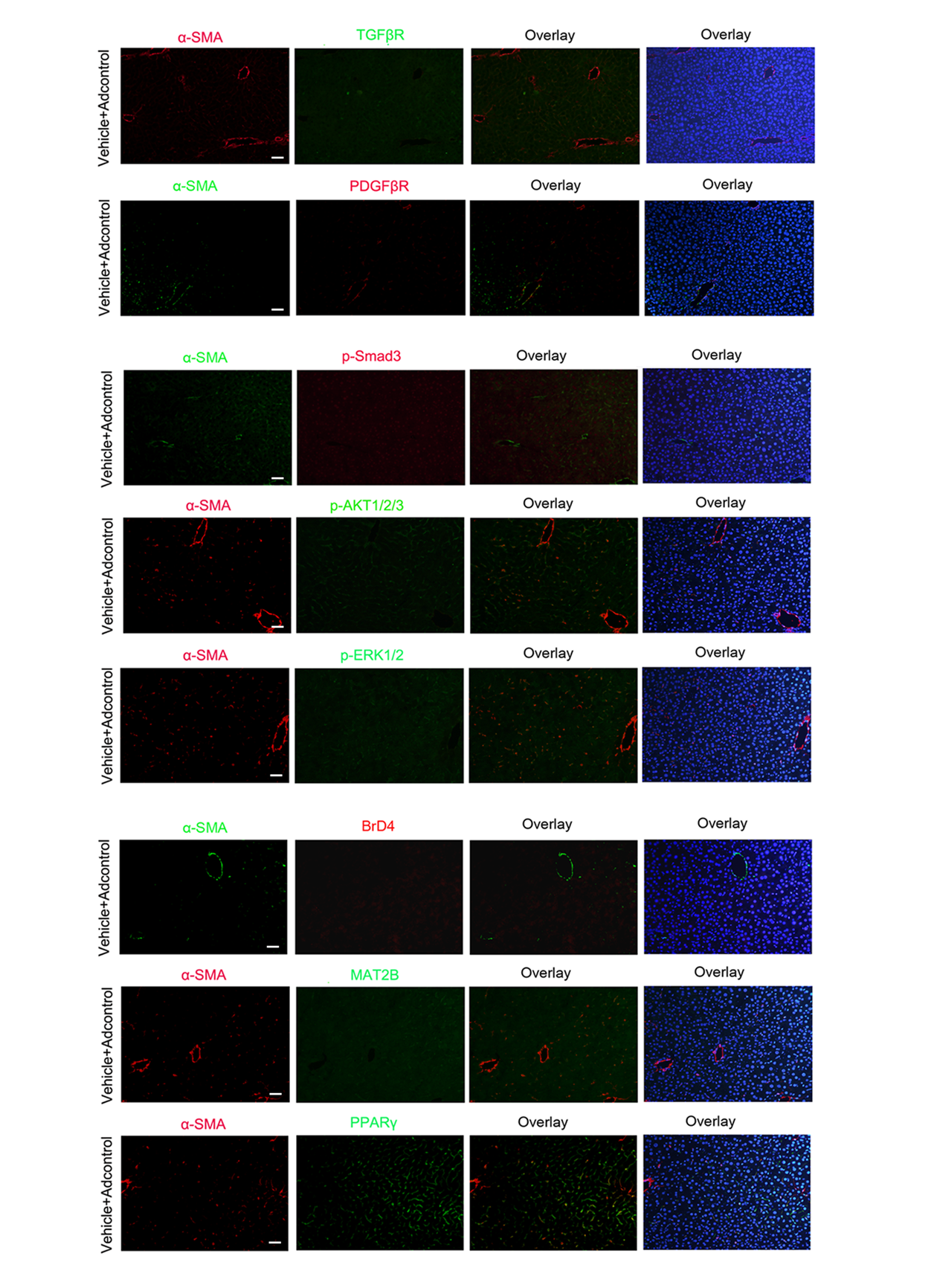


**Fig. S1. Double fluorescence staining of the livers.** The mice (six mice/each group) were received vehicle for 1 week and then were administered with vehicle plus Adcontrol (1×10^10^ pfu/mouse) for another 3 weeks by tail vein injection as described in Fig. 1C. Double fluorescence staining of the livers were performed for detecting α-SMA, TGFβR1, PDGFβR, phosphorylated Smad3, phosphorylated Akt1/2/3, phosphorylated ERK1/2, BrD4, MAT2B, and PPARγ in the livers. Scale bar: 50 μm.

# 9. Fig. S2. SREBP1c attenuates TAA-induced inflammation in liver.

#
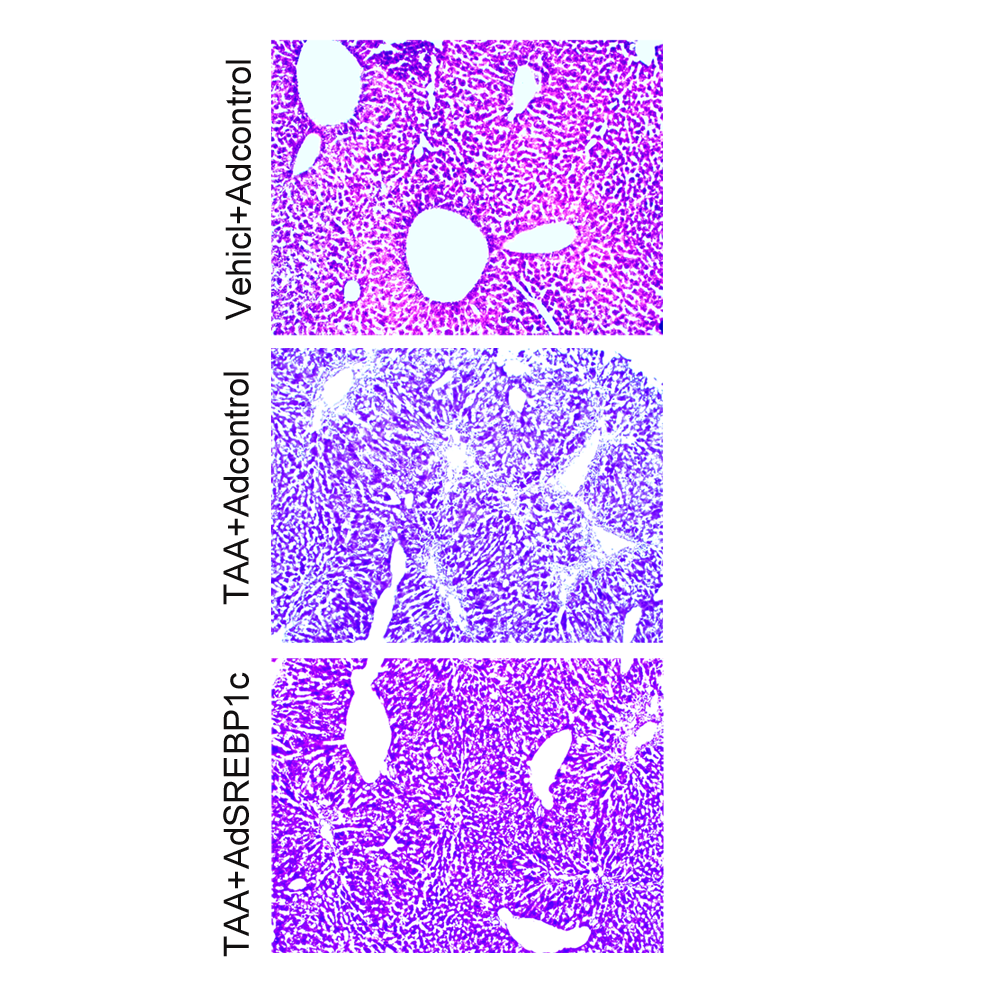


# Fig. S2. SREBP1c attenuates TAA-induced inflammation in liver. HE staining was performed on the liver sections from the same livers as in figure 1 by using Hematoxylin-Eosin (HE) Staining Kit (E607318, Sangon Biotech Co., Ltd. Shanghai, China) according to the manufacturer’s instructions. The inflammation was induced in the liver received TAA plus Adcontrol, which was attenuated by treatment with AdSREBP1c.

**10. Fig. S3. SREBP1c induces global DNA methylation in cultured HSCs.**


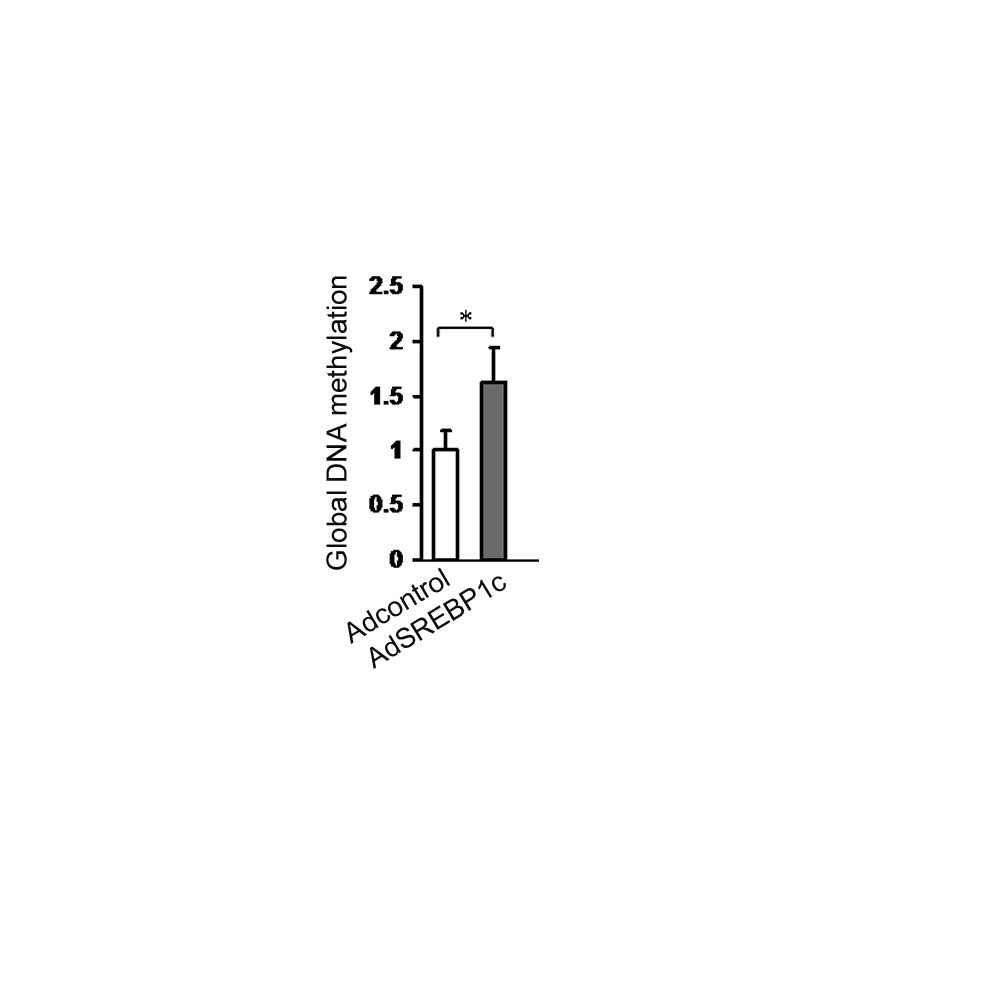


**Fig. S3. SREBP1c induces global DNA methylation in cultured HSCs (n = 3).** Culture-activated HSCs were infected with AdSREBP1c or the Adcontrol at 100 MOI for 48 h as described in figure 1E. DNA was isolated with the FitAmp Blood and Cultured Cell DNA Extraction Kit (P-1018, Epigentek Group Inc., N.Y., USA) according to the manufacturer's protocol and global DNA methylation in HSCs was determined by using the colorimetric MethylFlash Methylated DNA Quantification Kit (P-1035, Epigentek Group Inc., N.Y., USA) following the manufacturer’s instructions. p < 0.05.

**11. Fig. S4. Double fluorescence staining of GFP and α-SMA.**


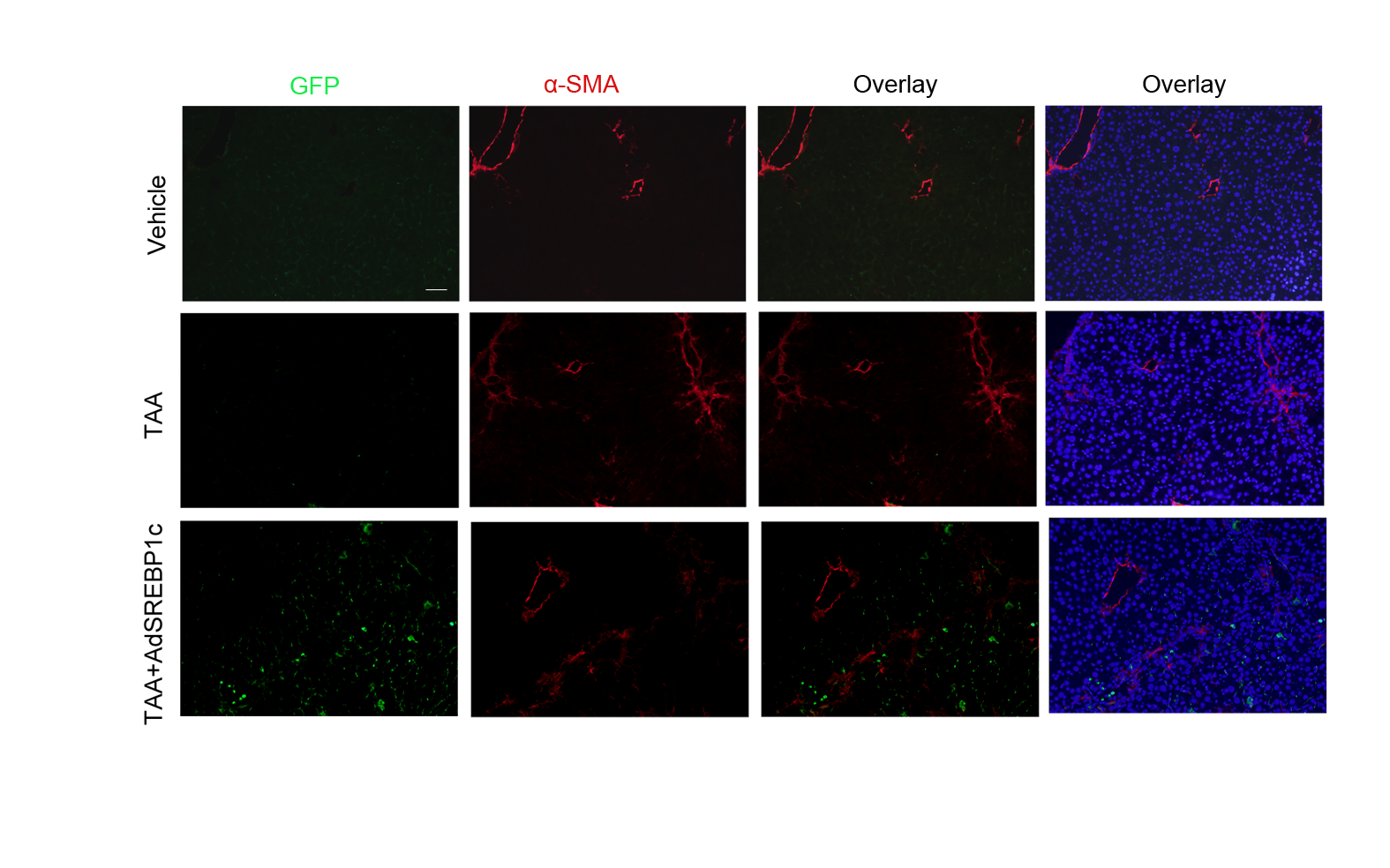


**Fig. S4. Double fluorescence staining of GFP and α-SMA.** Two groups of mice (six mice/each group) were received AdSREBP1c (1×10^10^ pfu/mouse) or vehicle by tail vein injection following one week of TAA treatment (200 μg/g body weight, three times a week, by i.p.) and then were treated with TAA for another three weeks. Double fluorescence staining of the liver sections was performed. The nuclei were counterstained with Hoechst 33342. Images were captured with light microscope. Scale bar: 50 μm.

Because there is not an appropriate marker for both quiescent HSCs and activated HSCs, we showed the activated HSCs by the antibody against α-SMA (1:250, ab124964, Abcam, MA, USA). In view that there are the endogenous SREBP1c in HSCs, we used the antibody against GFP (1:100, SC-9996, Santa Cruz, CA, USA) to indicate the expression of SREBP1c as the promoter in AdSREBP1c controls SREBP1c and GFP gene. If the AdSREBP1c express SREBP1c in HSCs undergoing activation, the HSCs will tend to be a quiescent state and thus quiescent HSCs can not be showed by fluorescence. So we can not provide a perfect result for its specificity by immunofluorescence. But the fluorescence for SREBP1c was demonstrated in the area of light red where there are more HSCs.
